# A scan for genes associated with cancer mortality and longevity in pedigree dog breeds

**DOI:** 10.1007/s00335-020-09845-1

**Published:** 2020-07-13

**Authors:** Aoife Doherty, Inês Lopes, Christopher T. Ford, Gianni Monaco, Patrick Guest, João Pedro de Magalhães

**Affiliations:** 1 Integrative Genomics of Ageing Group, Institute of Ageing and Chronic Disease, University of Liverpool, Liverpool, L7 8TX UK; 2 School of Biology, Medical and Biological Sciences Building, University of St. Andrews, North Haugh, St. Andrews, KY16 9TF UK

## Abstract

Selective breeding of the domestic dog (*Canis lupus familiaris*) rigidly retains desirable features, and could inadvertently fix disease-causing variants within a breed. We combine phenotypic data from > 72,000 dogs with a large genotypic dataset to search for genes associated with cancer mortality and longevity in pedigree dog breeds. We validated previous findings that breeds with higher average body weight have higher cancer mortality rates and lower life expectancy. We identified a significant positive correlation between life span and cancer mortality residuals corrected for body weight, implying that long-lived breeds die more frequently from cancer compared to short-lived breeds. We replicated a number of known genetic associations with body weight (*IGF1*, *GHR*, *CD36*, *SMAD2* and *IGF2BP2*). Subsequently, we identified five genetic variants in known cancer-related genes (located within *SIPA1*, *ADCY7* and *ARNT2*) that could be associated with cancer mortality residuals corrected for confounding factors. One putative genetic variant was marginally significantly associated with longevity residuals that had been corrected for the effects of body weight; this genetic variant is located within *PRDX1*, a peroxiredoxin that belongs to an emerging class of pro-longevity associated genes. This research should be considered as an exploratory analysis to uncover associations between genes and longevity/cancer mortality.

**Electronic supplementary material:**

The online version of this article (10.1007/s00335-020-09845-1) contains supplementary material, which is available to authorized users.

## Background

The World Health Organization predicts that the proportion of the world’s population over the age of 60 will nearly double from 12 to 22% between 2015 and 2050 (WHO 2016). Ageing is the biggest risk factor for cancer (de Magalhães 2013), which is a leading cause of deaths worldwide. For example, in 2018, approximately 18.1 million new cancer cases and 9.6 million cancer-related deaths were recorded globally (Bray et al. 2018). The biological complexity of human conditions such as cancer mortality and longevity has stimulated an intense search for experimental models that closely resemble the pathophysiological aspects of these processes (Kuningas et al. 2008; Alvarez 2014; de Magalhães 2014). As demonstrated previously for cancer and other traits (for example, Sutter et al. 2007; Paoloni et al. 2009; Pinho et al. 2012; Fenger et al. 2014; Schiffman and Breen 2015), the identification of novel genetic variants associated with complex conditions obtained from comparative mammalian models such as dogs has the potential to greatly advance our understanding of complex human conditions.

Over the last few centuries, approximately 193 American Kennel Club-recognised pedigree (i.e. purebred) dog breeds (*Canis lupus familiaris*) have evolved based on selection for particular characteristics that have been fixed and maintained by selective breeding within closed familial lines (AKC; Club 2009; Farrell et al. 2015). Artificial selection for specific traits circumvents Darwinian natural selection; although desirable features are rigidly retained, undesirable disease-causing gene variants risk being inadvertently increased in frequency or even fixed in the gene pool. For example, selective breeding can direct the enrichment of disease-causing alleles which would reflect in the high rates of specific diseases in some breeds, disease-causing mutations could hitch-hike with a desirable phenotypic trait, or there may be a pleiotropic effect of the selected variants (Sargan 2004; Karlsson and Lindblad-Toh 2008). In addition, the desired trait variant itself may lead to deleterious effects (Kanetsky et al. 2009). As a result of selective breeding, although a level of genetic heterogeneity is maintained between breeds (27% of total genetic variation compared to the typical 5–10% found in humans), some pedigree dog breeds, particularly those of European descent, have a reduced genetic diversity, as opposed to, for example, East Asian breeds (Parker et al. 2004; Freedman et al. 2014; Wang et al. 2016). This lower level of genetic variation (going up to 90% loss) has been seen before in several breeds, when their pedigree records are analysed, as well as an increase in inbreeding over time (Leroy et al. 2006; Calboli et al. 2008; Voges and Distl 2009; Jansson and Laikre 2014, 2018). The generation of pedigree dog breeds has proven to be a powerful genetic paradigm for the study of complex conditions such as longevity and cancer (Rowell et al. 2011; Ostrander 2012; Alvarez 2014; Schiffman and Breen 2015; Kaeberlein et al. 2016; Mazzatenta et al. 2017).

Canine malignancies have been established as exceptional comparative models for human cancers due to their similar spontaneous tumour development and frequency patterns, shared environment, similar risk factor exposure, response to conventional therapies, similar underlying genetics, and the high level of healthcare received by both species (Fleischer et al. 2008; Fleming et al. 2011; Ostrander 2012; Dobson 2013; Alvarez 2014; Ostrander et al. 2019). For example, using dogs as a model to study osteosarcoma has provided an unparalleled opportunity to understand its genetic drivers and the role of metastasis in the disease, and to pilot new investigatory drugs that would take too long to study in humans (Paoloni et al. 2009; Fenger et al. 2014).

From a longevity perspective, dogs age 5–8 times faster than humans and generally live to old age (Rowell et al. 2011; Kraus et al. 2013). Studies of longevity-associated genes in humans have generally been disappointing. With the exception of *APOE* (apolipoprotein E) (Christensen et al. 2006), few associations between specific gene loci and longevity have been replicated in multiple human populations. In contrast, there have been several robust genetic findings in model organisms of ageing (Partridge and Gems 2007; Kenyon 2010). For example, Sutter et al. (2007) mapped body weight to a single haplotype at the *IGF1* (insulin-like growth factor 1) locus that explained body weight variation both between and within breeds (Sutter et al. 2007); an observation that was repeated both in dogs (Jones et al. 2008; Akey et al. 2010; Boyko et al. 2010; Greer et al. 2011; Rimbault et al. 2013) and in other model organisms (Carter et al. 2002). This locus in dogs has also proven to be important in determining body weight and longevity in humans, as well as in other model organisms (Carter et al. 2002). These findings demonstrate that dogs are a suitable comparative genetic model, even for complex and poorly understood phenotypic traits such as longevity.

The aim of this experiment was to combine the recent availability of cancer mortality and longevity data for over 72,000 dogs from over 70 pedigree breeds (Fleming et al. 2011; Kraus et al. 2013) with a separate recently-published genotypic data set comprising over 166,171 single nucleotide polymorphisms (SNPs) from over 160 pedigree breeds (Shannon et al. 2015) to identify novel genetic variants that affect cancer mortality and longevity in pedigree dog breeds. Further investigation of these genetic variants has the potential to reveal new genes impacting canine and human health and longevity.

## Methods

### Data sources and quality filtering

Cancer mortality rates were derived from a large data set of 72,376 samples from 82 North American pedigree dog breeds (Fleming et al. 2011). Body weight and adult life expectancy (life expectancy at 4 years) data were obtained from 74 breeds comprising 56,637 samples (Kraus et al. 2013). Separately, a genotypic data set comprising 166,171 SNPs from 4676 pedigree dogs from 161 breeds was retrieved from Shannon et al. (2015). As the number of samples per breed ranged from 4 to more than 700 for the Labrador retriever (which is more than double the number of samples for the next highest breed) (Supplementary Table 1), we wanted to extract a roughly equal number of samples per breed. All samples from breeds with fewer than 30 samples were selected. For breeds with more than 30 samples representing the breed, 30 samples were selected at random to represent the breed; thus the population comprised 1274 samples; the number of samples per breed is now in line with previous similar analyses (Vaysse et al. 2011; Webster et al. 2015). 18,748 dog genes and their chromosomal locations were obtained from the *C. lupus familiaris* (CanFam 3.1) database in Ensembl BioMart version 82 (December 2015) (Cunningham et al. 2015). Breeds that possessed both phenotypic and genotypic data were extracted for further analysis; this left a combined data set of 63 breeds (Supplementary Table 1).

SNPs with a high level of missingness (> 10%), a low minor allele frequency (< 1%), located on sex chromosomes, or that were not in Hardy–Weinberg equilibrium among breeds (*P* < 0.01) were removed from the analysis using PLINK version 1.07 (Purcell et al. 2007). It is generally accepted that when SNPs are in strong LD, the alleles of some SNPs provide redundant information as the SNPs that they are in high LD with, as they tend to be inherited together. Consequently, a modest number of SNPs selected from each segment would suffice to define the relevant haplotypes in a population. For SNPs in high linkage disequilibrium (*r*^2^ > 0.9), one SNP in each pair was removed. Thus, the quality-filtered genotypic data set comprised 96,984 SNPs from 1274 samples from 63 pedigree dog breeds. We wanted to specifically focus our search on a set of candidate SNPs that were potentially related to longevity and cancer, as has previously been done many times to identify SNPs associated with complex traits (for example, Kulminski and Culminskaya 2013; Webster et al. 2015). Regarding longevity, 298 putative human ageing-related genes (https://genomics.senescence.info/genes/human.html; Supplementary Table 2a) and 1050 human homologs to model organism ageing-related genes (https://genomics.senescence.info/genes/models.html; Supplementary Table 2b) are collectively referred to as putative longevity-associated genes (LAGs) and were obtained from GenAge Build 17 (Tacutu et al. 2013). For more information on the assignment of longevity-associated genes, the reader is referred to (Tacutu et al. 2013). Model organism genes were converted to their respective human homologs and after removing redundancy, 903 LAGs remained (Supplementary Table 2c). These 903 LAGs, combined from human and human homologs to model organism ageing-related genes are referred to as the combined set LAGs. 803 dog genes with > 70% sequence identity to the combined set LAGs were retrieved from the Ensembl BioMart database (Cunningham et al. 2015) (Supplementary Table 2d). The chromosomal location of each gene was identified using the CanFam 3.1 assembly in Ensembl BioMart (Supplementary Table 2e), and 2874 of the quality-filtered SNPs located within ± 20 kb of each gene were identified as putatively longevity-associated and extracted for further analysis (Supplementary Table 2f). For the cancer mortality analysis, 352 genes in the dog KEGG pathway “cfa05200—Pathways in cancer” with Ensembl IDs were obtained from (Kanehisa 2000) (Supplementary Table 2g), and 1544 SNPs located within ± 20 kb of these genes were retained for further analysis (Supplementary Table 2h). The interval ± 20 kb was selected based on LD decay found in the work of Boyko et al. (2010). This value, while not completely conservative, it is also not found on the end of the LD decay curve, allowing us to detect SNPs in close proximity and in association with the genes here studied.

### Spearman correlation to assess association between cancer mortality, body weight and life expectancy

Spearman’s rank correlation tests were conducted to assess the associations between cancer mortality rates, body weight, and life expectancy using SciPy version 0.16.1 (Jones et al. 2014) in Python version 2.7.3. For the correlation analysis (i.e. the Results section entitled “Correlation between cancer mortality, body weight, and life expectancy”), there was no significant correlation observed between cancer mortality rates and life expectancy rates. To understand if the same effect would be observed if we considered cancer mortality rate residuals after correction for body weight, we conducted a linear regression between cancer mortality rates and body weight using the “linregress” function SciPy version 0.16.1.

For the association analysis between cancer mortality/longevity-related SNPs and cancer mortality/longevity, to permit associations with cancer mortality rates to be investigated independently from body weight and life expectancy, standardised residuals for the cancer mortality rates were calculated after a multiple regression analysis with body weight and life expectancy calculated using StatsModels version 0.6.1 (Seabold and Perktold 2010). In addition, to permit association with longevity data to be investigated independently from body weight, standardised residuals for longevity were calculated after a linear regression analysis using SciPy version 0.16.1.

Additionally, in order to access the effect of outliers, we jackknifed the correlation coefficients obtained in the Spearman tests using the jackknife package version 2019.6 and R version 3.5.2.

### Association analysis between SNPs of interest and phenotypic traits (i.e. body weight, cancer mortality or longevity)

PLINK version 1.07 (Purcell et al. 2007) was used to conduct a linear association analysis with the commonly implemented additive genotypic model (Clarke et al. 2011). Three association analyses were conducted between (1) 96,984 quality-filtered SNPs and body weight, (2) a set of 1544 cancer-associated SNPs and cancer residuals (i.e. after multiple regression with body weight and life expectancy) and (3) a set of 2874 longevity-associated SNPs and longevity residuals (i.e. after linear regression with body weight). Corrected *P* values were calculated using the permutation procedure as described in (Vaysse et al. 2011). This procedure has previously been used successfully in studies such as (Karlsson et al. 2007; Vaysse et al. 2011; Webster et al. 2015) to both replicate others’ findings, and to identify novel genetic variants that are associated with various phenotypic traits. Briefly, we used a breed-specific permutation procedure to determine genome-wide significance implemented using a Python (version 2.7.3) script. Each sample within a breed was assigned a phenotype corresponding to the breed-specific value of a trait. Traits were coded as quantitative in these experiments (i.e. mortality residual and longevity residual data). An association study was performed for each trait followed by a permutation procedure, in which the phenotypes of each breed were randomised and identical phenotype values were always assigned to each sample within the same breed. For each experiment, 1000 permutations were performed. We identified the lowest *P* value (i.e. the lowest permuted *P* value) obtained across all of the SNPs in each of the 1000 permutations. Then, we calculated how many times our raw *P* value was lower than the lowest permuted *P* value using the equation (1 + # times raw *P* Value was lower than the lowest permuted *P* values/1001). In this way, the raw significance value of each SNP was compared to the minimum permuted *P* value across all SNPs to calculate corrected significance values. SNPs that obtained a corrected *P* < 0.05 were considered significant. This permutation process corrects for the extreme population sub-structure present in dog breeds. *P* values are calculated across all of the SNPs used in each analysis. In regards to the correlation analysis with minor allele frequency (MAF), the Pearson and Spearman values were obtained using SciPy version 0.16.1 and the FDR was calculated using the Benjamini & Hochberg method.

The robustness of the identified SNPs of interest was tested using a resampling method for the two analyses of interest (i.e. cancer mortality residuals after multiple regression with body weight and life expectancy and longevity residuals after linear regression with body weight). The data were subsampled 10 times, randomly removing 10% of the genotypic subjects each time (samples remaining = 1147). As can be observed from Supplementary Tables 4b and 5b, the number and identities of SNPs significantly associated with both traits per chromosome remained relatively constant across the original and resampled data sets, suggesting that the associations are robust and not dependent on the exact set of samples used in the analysis.

## Results

### Correlation between cancer mortality, body weight, and life expectancy

**Fig. 1:**
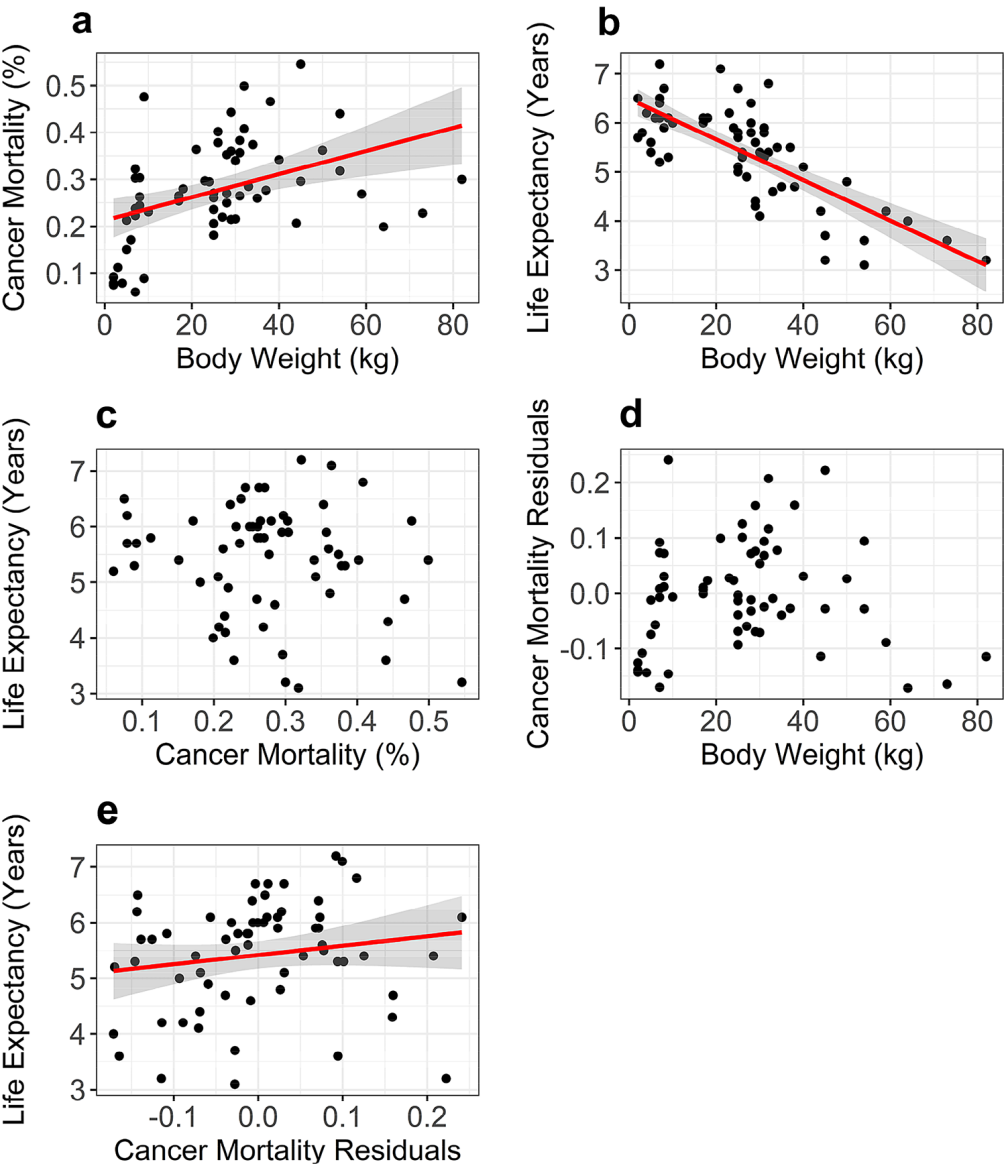
Correlation results for cancer mortality, body weight and life expectancy. **a** Raw cancer mortality (i.e. cancer mortality rate before linear regression with body weight) versus body weight (Spearman correlation, *R* = 0.5; *P* = 2.88E−05; Linear Regression, *R* = 0.413; *P* = 7.7E−04). **b** Life expectancy versus body weight (Spearman correlation, *R* = − 0.68; *P* = 6.73E−10). **c** Life expectancy versus raw cancer mortality (i.e. cancer mortality rate before linear regression with body weight) (Spearman correlation, *R* = − 0.06; *P* = 0.63). **d** Cancer mortality residuals after linear regression with body weight versus body weight. **e** Life expectancy versus cancer mortality residuals after linear regression with body weight (Spearman correlation, *R* = 0.24; *P* = 0.05)

For 63 dog breeds (*N* = 1274 dogs; Supplementary Table 1), a data set comprising body weight, life expectancy and cancer mortality rates was assembled and quality filtered. Concordant with prior studies (Fleming et al. 2011; Dobson 2013), we found a strong significant positive correlation between cancer mortality and body weight in our dog breeds (*R* = 0.5, *P* = 2.88E−05, Spearman’s Test; $\hat{R}=0.51,$ Jackknife correlation; Fig. 1a). Additionally, in agreement with previous studies (Fleming et al. 2011; Kraus et al. 2013), a strong, significant, negative correlation between body weight and life expectancy was observed (*R* = − 0.68, *P* = 6.73E−10, Spearman’s test; $\hat{R}=-0.69,$ Jackknife correlation; Fig. 1b). There was no significant association between the raw cancer mortality data and life expectancy (*R* = − 0.06, *P* = 0.63, Spearman’s Test; $\hat{R}=-0.06,$ Jackknife correlation; Fig. 1c). Given the strong correlation between cancer mortality and body weight, and to avoid finding genetic associations with cancer merely due associations with body weight, a linear regression between body weight and cancer mortality was conducted to obtain cancer mortality residuals that were corrected for the effects of the correlation between cancer mortality and body weight (Fig. 1a and d). There was a moderate significant positive correlation between life expectancy and cancer mortality residuals after linear regression with body weight (*R* = 0.24, *P* = 0.05, Spearman’s Test; $\hat{R}=0.25,$ Jackknife correlation; Fig. 1e). Overall, the bias-corrected jacknife estimates values ($\hat{R}$) were very similar to the correlation coefficients, showing that outliers had little influence in correlations here observed.

### Genetic association analysis for body weight

We performed a quantitative association analysis between all 96,984 SNPs in the data set and body weight for 63 breeds with phenotypic and genotypic data (*N* = 1274 dogs), and analysed the statistical significance of each association using a strict permutation procedure for two reasons. First, there are a number of genetic variants that are well known to be associated with body weight both in dogs, and in other organisms. The replication of these observations in our population would serve as validation that our genetic association and permutation method was capable of identifying phenotypically relevant genetic variants. In addition, both of our main traits of interest, longevity and cancer mortality, are significantly correlated with body weight. Should any SNPs be found to be associated with cancer mortality or longevity, we aimed to understand if such associations are artefacts of an association with body weight. The full output from the association analyses between all 96,984 SNPs and body weight, along with the corrected *P* values for each SNP from the permutation analysis is found in Supplementary Table 3a. To check for robustness of the observations, the data set was resampled (Supplementary Table 3b). To validate our observations further, we conducted a Spearman correlation between per-breed body weight and per-breed minor allele frequency, for all 96,984 SNPs.

**Fig. 2:**
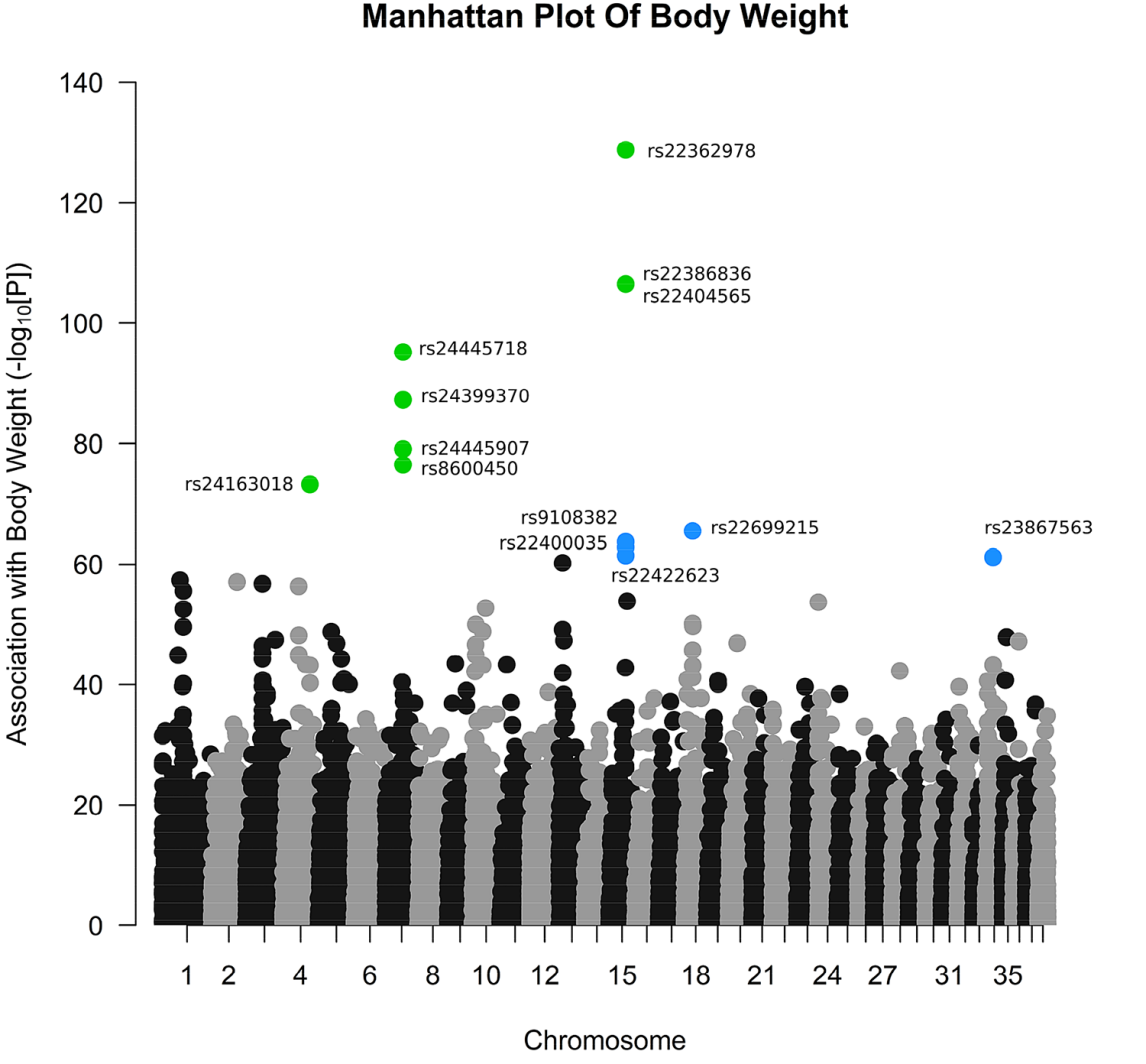
Manhattan Plot of associations between SNPs and body weight. The points coloured green were significantly associated with body weight after permutation and the points coloured blue were marginally significant with body weight after permutation. The points coloured black/grey (they are indicative of alternating chromosomes) are SNPs not associated with body weight after permutation

There were eight SNPs significantly (corrected *P* < 0.05) and a further six SNPs marginally significantly (corrected *P* < 0.1) associated with body weight from the GWAS and resampling analyses (Supplementary Table 3; Fig. 2); the majority of these SNPs are also significant in the Spearman correlations between MAF and trait (Supplementary Table 6a). From the European Variation Archive (EVA) (EMBL-EBI), we observed that six of the fourteen SNPs (rs22362978, rs22386836, rs22404565, rs9108382, rs22400035 and rs22422623) are within intron regions of the gene insulin growth factor 1 (*IGF1*). One SNP (rs24163018) is within an intron region of growth factor receptor (*GHR*) and one SNP (rs22699215) is within an intron region of cluster of differentiation 36 (*CD36*). A further two SNPs (rs24445718 and rs24445907) are in close proximity (i.e. ± 20 kb) to the SMAD Family Member 2 (*SMAD2*) gene and one SNP (rs23867563) is within close proximity to the insulin-like growth factor 2 mRNA binding protein 2 (*IGF2BP2*) gene (Supplementary Table 3).

### Three SNPs associated with cancer mortality in pedigree dog breeds

To identify genetic variants that are associated with cancer mortality in pedigree dog breeds, we conducted a targeted approach to genetic variant-trait association discovery, similar to studies that have previously successfully identified SNPs associated with various complex traits (for example, Willcox et al. 2008; Kulminski and Culminskaya 2013; Webster et al. 2015). A total of 352 genes in the dog KEGG pathway “cfa05200—Pathways in cancer” were retrieved from (Kanehisa 2000), and 1544 of the quality filtered SNPs located within ± 20 kb of these genes were extracted (see “Methods” section for a more detailed description of the SNP selection process). We performed a quantitative association analysis between these 1544 potentially cancer-related SNPs and cancer mortality residuals after multiple regression for body weight and life expectancy in the 63 breeds for which both phenotypic and genotypic data was available (*N* = 1274 dogs; see “Methods” section). The full association results for all of the SNPs, and the results of the resampling analysis, are found in Supplementary Table 4a and 4b.

**Fig. 3:**
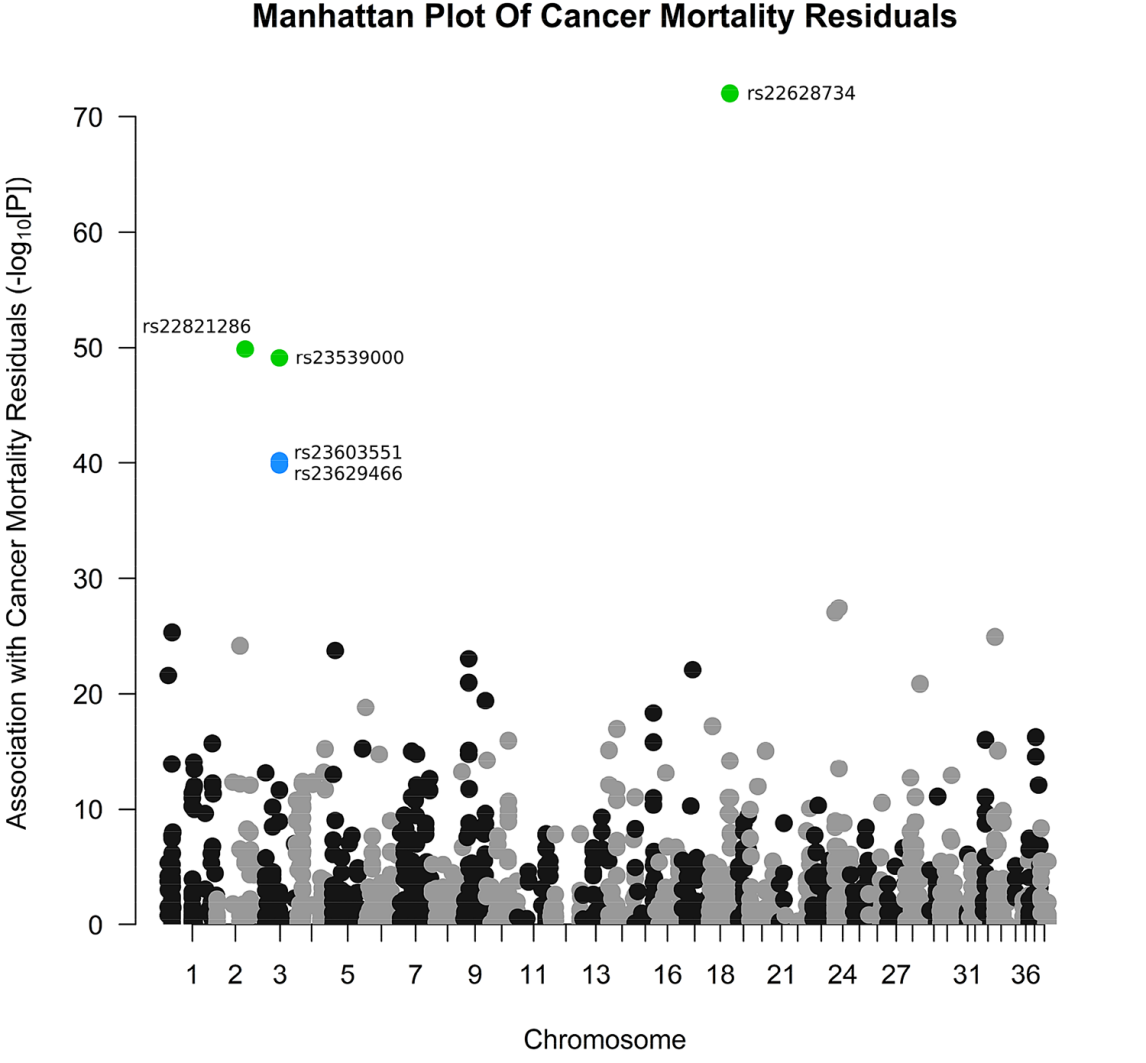
Manhattan Plot of association between SNPs and cancer mortality residuals, after multiple regression with life expectancy and body weight. The points coloured green were significantly associated with cancer mortality residuals after permutation and the points coloured blue were marginally significant with cancer mortality residuals after permutation. The points coloured black/grey (that are indicative of alternating chromosomes) are SNPs not associated with cancer mortality residuals after permutation

After correction for body weight, only three SNPs (rs22628734, rs23539000 and rs22821286) were statistically significantly associated with cancer mortality residuals (corrected *P* < 0.05); and a further two SNPs (rs23603551 and rs23629466) were marginally significantly associated with cancer mortality residuals (corrected *P* < 0.1) (Table 1; Supplementary Table 4a, Fig. 3). Encouragingly, these five SNPs were consistently significant or marginally significant in the set of resampled data sets (Supplementary Table 4b). Three of the SNPs (rs23539000, rs22821286 and rs23603551) had uncorrected *P* values that were not significantly associated with body weight, and all of the SNPs were not significantly associated with body weight after permutation correction (Supplementary Table 6b). Only rs23539000 was in high LD (*r*^2^ > 0.9) with one other SNP in the original pre-quality filter data set comprising 166,171 SNPs obtained from (Shannon et al. 2015) (rs23589426, *r*^2^ = 0.91). To attempt to validate our observations using an independent method, we conducted a Spearman correlation between the per-breed MAF for all 1544 SNPs and cancer mortality residuals. The 30 SNPs that obtained the lowest correlation between minor allele frequency and trait is found in Supplementary Table 6c. Two of the SNPs identified using the GWAS approach (rs23629466 and rs23539000) exhibit pre-FDR *P* values < 0.05 from the Spearman correlation approach; and the strength of the correlations themselves are relatively weak (*R* < 0.35; Supplementary Table 6b). However, given the fact that we are attempting to identify SNPs associated with a relatively complex trait, it is possible that this independent method would not identify our SNPs of interest as easily as for other traits, such as body weight as described in a previous section.

**Table 1:** Single nucleotide polymorphisms associated with cancer mortality residuals and longevity residuals under the additive genotypic model

Data set	Chr	Position (base pairs) according to CanFam 3.1	SNP name (rs #)	Genes that SNPs are in	Association with residuals; corrected *P* value (* = marginal significance)	Association with body weight; corrected *P* value
Cancer mortality residuals	2	65,016,323	rs22821286	*ADCY7*	0.02	1.0
	3	57,088,297	rs23629466	*ARNT2*	0.08*	1.0
	3	57,118,495	rs23539000	*ARNT2*	0.02	1.0
	3	57,122,649	rs23603551	*ARNT2*	0.07*	1.0
	18	51,567,888	rs22628734	*SIPA1*	0.002	1.0
Longevity residuals	13	50,802,666	rs9067088	–	0.08*	1.0

SNPs of interest were searched for in the EVA (EMBL-EBI) database to identify their functional consequence on protein structure and function. All of these SNPs are located within the intron regions of three genes: rs22628734 is located in signal-induced proliferation-associated 1 (*SIPA1*), rs23629466, rs2359000 and rs2360351 are located in aryl hydrocarbon receptor nuclear translocator 2 (*ARNT2*) and rs22821286 is in adenylate cyclase 7 (*ADCY7*) (Supplementary Table 6b).

### One genetic variant on chromosomes 13 is potentially associated with longevity residuals in pedigree dog breeds

**Fig. 4:**
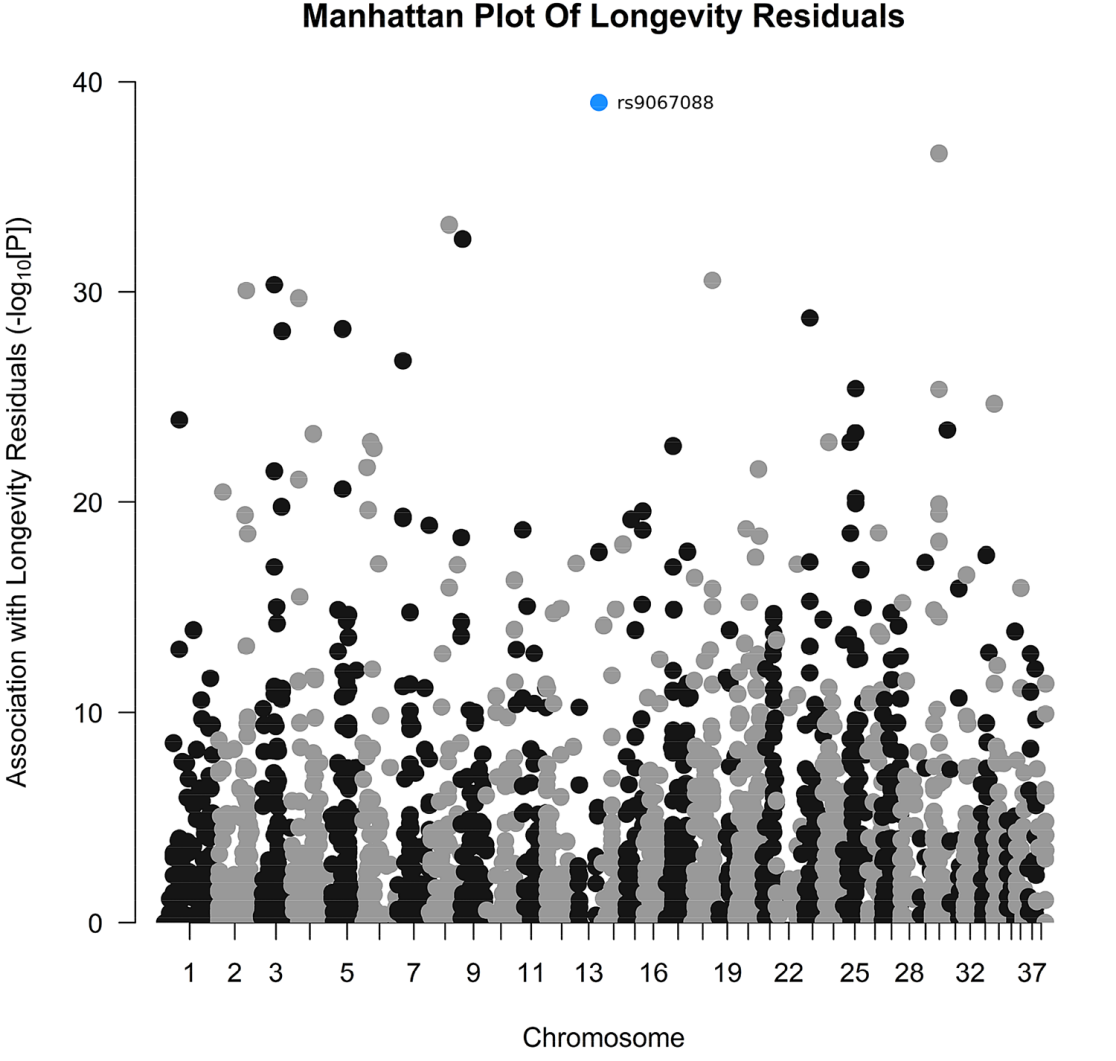
Manhattan plot of association between SNPs and longevity residuals, after linear regression with body weight. The point coloured blue was marginally significant with longevity residuals after permutation. The points coloured black/grey (that are indicative of alternating chromosomes) are SNPs not associated with longevity residuals after permutation

Similar to the cancer mortality analysis, a targeted candidate gene approach was conducted to identify SNPs and genes associated with longevity. A data set comprising 803 genes that are putatively related to ageing in dogs was assembled (Supplementary Table 2d). 2,874 quality-filtered SNPs located within ± 20 kb of each potential ageing-related gene were identified (Supplementary Table 2f). We performed an association analysis between these 2,874 potentially ageing-related SNPs and longevity residuals (i.e. after regression between longevity and body weight) using the same 1,274 samples described in the previous section (see “Methods” section). The results from the full association analysis are in Supplementary Table 5a and 5b. There was one SNP of interest (rs9067088) that was marginally significantly associated with longevity residuals (Corrected *P* = 0.09; Table 1; Supplementary Table 5b; Fig. 4). Rs9067088 is not associated with body weight after permutation and is not in high linkage disequilibrium with any other SNP in the data set (Supplementary Table 6b). We conducted a Spearman correlation between the MAF of all 2874 SNPs and longevity residuals. The 30 SNPs displaying the 30 lowest pre-FDR Spearman correlations is found in Supplementary Table 6d. Our SNP of interest did not demonstrate a significant correlation between per-breed MAF and longevity residuals (Supplementary Table 6b); and is not represented in the 30 lowest correlations between MAF and longevity residuals; however given that longevity is such a complex trait, perhaps a simple Spearman correlation is not sensitive enough to pick up such subtle genetic effects.

This SNP lies in a homolog to the human peroxiredoxin 1 (*PRDX1*) gene, and is assigned to the Ensembl gene family “PTHR10681_SF75”; a peroxiredoxin (Prx)-related gene family that contains *PRDX1* homologs from numerous other species.

### Discussion

Although cancer and longevity are multifactorial processes influenced by complex genetic and environmental factors, the fact that, among the pedigree dog breeds, both cancer incidence and lifespan vary significantly suggest that genetic mechanisms are involved. Furthermore, such variation can be detected and compared, as there is artificially enriched variation between pedigree breeds and relative homogeneity within breeds. Genetic association using breed-level clinical and genetic data therefore offers a powerful approach to discover genetic variants underlying canine cancer and longevity with potential human applications (Sutter et al. 2007; Paoloni et al. 2009; Ostrander 2012; Alvarez 2014; Fenger et al. 2014; Schiffman and Breen 2015). We combined a large, recently-generated genotypic data set from over 1500 dogs (Shannon et al. 2015) with a cancer, longevity and body weight data set (Fleming et al. 2011) encompassing the same breeds. Our aim was to locate predisposing genes across many breeds that affect these complex conditions, while using a permutation procedure that corrects for the extreme population sub-structure present in dog breeds. Finally, we considered the potential molecular mechanisms through which such genetic variants could exert their effect.

A number of correlations between phenotypic traits have previously been described in the domestic dog. For example, in this work we replicate the previously described negative correlation between body weight and lifespan and the positive correlation between body weight and cancer mortality rate (Fleming et al. 2011; Nunney 2013; Kraus et al. 2013; Song et al. 2013). The latter is in direct contrast with the Peto’s Paradox, which states that, because of body mass, large animals should be more susceptible to cancer, which is rare in a lot of big long-lived animals (like the elephant or the bowhead whale) (Tollis et al. 2017). Additionally, in our data set, although raw cancer mortality rate (i.e. before multiple regression with life expectancy and body weight) was surprisingly not correlated with breed lifespan, cancer mortality residuals after linear regression with body weight showed a significant positive association with lifespan, i.e. long-lived breeds appeared to die more frequently from cancer than would be expected when differences in body weight are controlled for. Given the strong epidemiological association of cancer with age (Anisimov 2003; de Magalhães 2013), the observed relationship between life expectancy and cancer mortality residuals may reflect the established multistep model of cancer. In this scenario, alterations to various biological systems occur with ageing that affect cancer incidence and progression, independent of body weight.

We first conducted a linear genetic association study between all 96,984 SNPs in the data set with body weight. Several studies have performed multi-breed GWAS for body weight in dogs. In spite of variation in breeds and markers used in each data set, some key observations from previous studies are consistent with our analysis. The majority of the fourteen SNPs of interest that were significant or marginally significant after the permutation analysis are in genes that are well-known for their association with body weight, including *IGF1*, *GHR*, *CD36*, *SMAD2* and *IGF2BP2*, all of which have clearly been demonstrated to affect body weight in multiple organisms (Baker et al. 1993; Liu et al. 1993; Efstratiadis 1998; Sims et al. 2000; Pravenec et al. 2001; Carter et al. 2002; Mohan et al. 2003; Sutter et al. 2007; Jones et al. 2008; Chase et al. 2009; Akey et al. 2010; Boyko et al. 2010; Greer et al. 2011; Rimbault et al. 2013; Plassais et al. 2019). These results were validated using an independent approach that correlated the minor allele frequency of each breed with the per-breed body weight. These observations provided proof of concept that a quantitative genetic association analysis followed by a strict permutation procedure selects phenotypically relevant genetic variants for the trait of interest.

We examined 1544 SNPs that potentially affect cancer mortality due to their proximity to known cancer-related genes. In total, we found five genetic variants that were significantly associated with cancer mortality after permutation, were not significantly associated with body weight after permutation (and the majority were also not significantly associated with body weight before permutation), and were also observed as significant in the resampled data set. All of these SNPs are located within three genes: signal-induced proliferation-associated 1 (*SIPA1*), aryl hydrocarbon receptor nuclear translocator 2 (*ARNT2*) and adenylate cyclase 7 (*ADCY7*) (Supplementary Table 6); genes that have been repeatedly demonstrated to be associated with tumour growth and cancer prognoses for numerous cancer types (Park et al. 2005; Crawford et al. 2006; Minato and Hattori 2009; Hsieh et al. 2009; Brooks et al. 2010; Yang et al. 2015; Li et al. 2015a, b; Kimura et al. 2016). It is worth noting that, while there exist several studies that tried to identify loci associated with cancer within breeds (Phillips et al. 2010; Shearin et al. 2012; Karyadi et al. 2013; Tonomura et al. 2015; Melin et al. 2016; Hayward et al. 2016), there is a lack of studies among breeds. Two such studies found that there was no significant association across breeds (for osteosarcoma and lymphoma) (Karlsson et al. 2013; Hayward et al. 2016), and another found several loci strongly associated with glioma across breeds (Truvé et al. 2016).

Focusing on a set of ageing-related SNPs, we found a marginally significant association between one SNP and longevity residuals that were corrected for body weight in pedigree dog breeds in the original analysis, and also in the majority of resampled data sets. This SNP lies in a novel dog gene (*ENSCAFG00000002337*) that is a homolog to the human peroxiredoxin 1 (*PRDX1*) gene. This gene does not appear to be regularly discussed in relation to body weight, but has previously been demonstrated to be important for protection against apoptosis and oxidative stress, and the promotion of longevity in a range of organisms (Lee 2003; Olahova et al. 2008; Radyuk et al. 2009; Nystrom et al. 2012; De Haes et al. 2014). It should be noted, that this SNP was only marginally significant in its association with longevity, and even after further permutation the significance was not fully reflected. Further experimental validation might be required in order to assess whether there is a significant association between this gene and longevity or if this was a false-positive observation.

There are a number of limitations to be considered in this preliminary analysis. Due to the fact some breeds used in this study were underrepresented, the ability to detect associations for these cases may be flawed. Body weight was an average value for each breed and not a value obtained for each individual sample used, which means that this study did not account for body weight variability between individuals and its impact in the detection of associations. On top of that, cancer mortality and life expectancy were obtained from previous studies that used the Veterinary Medical Database (VMDB) as their data source. The VMDB compiles data from several member hospitals in North America and it only represents a subsample of the whole dog population which can introduce some bias, specifically due to the seriousness of the diseases recorded, the possibility of misdiagnosis or misclassification of breeds, as noted by Fleming et al. (2011). Additionally, since the records pertain to veterinary practices, there is a bias towards non-healthy dogs, which might mean that some breed longevities were underestimated. The inability to infer the exact mechanisms through which these genetic variants could influence longevity and cancer mortality is unsurprising, given the complex nature of both of these traits, the known difficulties of identifying genes associated with longevity even in much larger human studies and the nature of this experiment (de Magalhães 2014). As clarified earlier, the associations described should be considered putative, given that the same observation was not made using two independent methods (possibly due to the subtlety of the traits) and the fact that there is no previous literature available that identifies similar associations between this SNP and longevity-related traits. We stress that it will be essential to attempt to validate all cancer mortality and longevity residual findings in this research, once a suitable population arises. We also note that, while it is possible that the SNPs here identified contribute to the phenotypes studied, a whole genome sequence analysis would be necessary to determine whether there are better casual variants than the variants acknowledged in this work. Experimental validation should also be done in an attempt to infer the mechanisms associated with these findings. Unfortunately, due to the present scarcity of high quality, large-scale dog SNP genotype datasets, such a replication analysis was not feasible at this time. In addition, it is not currently possible to assess the individual contributions of distinct cancer subtypes to the associations we observed between cancer mortality and SNPs, or the mechanism through which each genetic variant is exerting an effect. Indeed, it is possible that the SNPs found to influence cancer mortality and longevity are exerting effects on multiple traits (i.e. pleiotropy), a scenario that is not examined in detail in this research. Furthermore, while this analysis was able to detect associations between both cancer mortality and longevity with SNPs across all breeds, if one breed has a particular cancer risk SNP or longevity protective SNP that is unique to it, it’s very likely that such association was not properly detected within the confines of this study. Similarly, for breeds with a great predisposition to life-threatening diseases, the effects of longevity protective SNPs may have not been observed due to their untimely death.

That being said, the identification of even a single putative SNP that is potentially associated with longevity requires further attention, given the difficulty in identifying SNPs associated with this trait generally. This research provides a starting point for additional studies to validate the results described herein, once such data become available.

### Conclusion

Over the last two decades, the domestic dog has emerged as a powerful genetic paradigm for the study of heritable human disease due to the evolutionary history of pedigree dog breeds, the high level of healthcare they typically receive and their genetic and pathophysiological similarities to humans. Using this comparative model has advanced our understanding of a number of human diseases and potential therapies, such as disorders associated with immunodeficiency, cancer, and metabolic disease. In particular, furthering our understanding of the molecular mechanisms responsible for cancer and longevity in dogs will aid drug development in the comparable human conditions. This investigation identified genetic variants associated with cancer mortality and longevity. Although we were able to replicate some previous findings for genetic associations between SNPs and body weight, and also validate these findings using two independent methods, the same cannot be said for the putative genetic associations that were identified between SNPs and cancer mortality/longevity. Thus, we stress that this analysis should be considered exploratory and the results considered as indicative, and we suggest that further studies are warranted to confirm these associations and further explore the relationships of the identified genes with cancer and longevity in dogs and humans.

## Electronic supplementary material

Below is the link to the electronic supplementary material.

Supplementary file 1 (XLSX 15 kb)

Supplementary file 2 (XLSX 1041 kb)

Supplementary file 3 (XLSX 8678 kb)

Supplementary file 4 (XLSX 149 kb)

Supplementary file 5 (XLSX 254 kb)

Supplementary file 6 (XLSX 20 kb)

## Data Availability

All data resulting from this work is available publicly in the Supplementary Material at Github (https://github.com/maglab/dog-supplementary). All of the SNP data is available publicly from https://datadryad.org/resource/doi:10.5061/dryad.v9t5h.
